# Antibody-Conjugated Silica-Modified Gold Nanorods for the Diagnosis and Photo-Thermal Therapy of *Cryptococcus neoformans*: an Experiment In Vitro

**DOI:** 10.1186/s11671-018-2487-4

**Published:** 2018-03-07

**Authors:** Qin Xiao, Yongzhou Lu, Min Chen, Bo Chen, Yuming Yang, Daxiang Cui, Bo Pan, Nan Xu

**Affiliations:** 1 Department of Dermatology, Shanghai East Hospital, Tongji University, Shanghai, China; 2 Department of Dermatology, Changzheng Hospital, Second Military Medical University, Shanghai, China; 3 Department of Radiology, Shanghai East Hospital, Tongji University, Shanghai, China; 4 Institute of Nano Biomedicine and Engineering, Shanghai Jiao Tong University, Shanghai, China

**Keywords:** Gold nanorods, *Cryptococcus neoformans*, Diagnosis, Photo-thermal therapy

## Abstract

**Background:**

*Cryptococcus neoformans* is an encapsulated yeast. There is still little quick and effective solution for the diagnosis or treatment of *C. neoformans* infection at an early stage in clinical. Antibody-conjugated silica-modified gold nanorods (GNR-SiO_2_-Ab) can conjugate *C. neoformans* selectively. It may provide a possibility for treatment of cryptococcosis safely and effectively.

**Methods:**

Gold nanorods (GNRs) were synthesized according to the seed-mediated template-assisted protocol. Anti-*C. neoformans* antibody was covalently anchored on the surface of GNRs with silane coupling agent. In vitro computer tomography imaging was performed to explore the diagnostic effect of the GNR-SiO_2_-Ab. The viability of cells was evaluated to confirm the photo-thermal therapy effect of GNR-SiO_2_-Ab combined with near-infrared (NIR) laser light.

**Results:**

GNR-SiO_2_-Ab has a potential application as a positive X-ray/CT imaging contrast agent. An antibody can induce a much greater aggregation of GNRs by binding to the surface of *C. neoformans* cells resulting in a much higher attenuation values than ever. After irradiation, *C. neoformans* cells suffered photo-thermal damages and the normal structure of cells were destroyed. The viability of cells reduced significantly compared to the untreated cells.

**Conclusions:**

Our work confirmed that antibody-conjugated silica-modified gold nanorods could enhance X-ray attenuation of *C. neoformans* cells in CT images. And immune GNRs, which were mediated by antibodies, could increase the effects of NIR-induced photo-thermal therapy in *C. neoformans* cells.

## Background

*Cryptococcus neoformans* is an encapsulated yeast, which was first described by Busse in 1894 [1]. Infection with the encapsulated yeast *Cryptococcus neoformans* can result in harmless colonization of the airways, but it can also lead to meningitis or disseminated disease [2], especially in persons with defective cell-mediated immunity. Cryptococcosis represents a major life-threatening fungal infection in patients with severe HIV infection and may also complicate organ transplantation, reticuloendothelial malignancy, corticosteroid treatment, or sarcoidosis [3]. Cryptococcal meningitis associated with HIV infection is responsible for more than 600,000 deaths per year worldwide [4]. Cryptococcal meningitis and disseminated disease were invariably fatal. In 1995, Speed and Dunt reported a 14% mortality rate among patients with cryptococcal disease who were treated with amphotericin B plus flucytosine [5]. The workup in patients with suspected cryptococcosis depended on fungal culture. However, there is still little quick and effective solution for the diagnosis or treatment of *C. neoformans* infection at an early stage. In addition, most patients with Cryptococcal infections fail to receive the prompt treatments, resulting in a high mortality rate.

Among all the imaging techniques, X-ray computed tomography (CT) is one of the most useful diagnostic tools in hospitals in terms of availability, efficiency, and cost [6]. CT is able to identify anatomical patterns and to provide complementary anatomical information including tumor location, size, and spread on endogenous contrast [7]. One common manifestation of pulmonary cryptococcosis is the presence of solitary or multiple pulmonary nodules or masses, cavitation, or parenchymal abnormalities. These manifestations are clearly detected by computerized tomography (CT) imaging [8]. Using radiographic imaging, the following features of *Cryptococcal meningitis* are typically presented: dilated Virchow-Robin spaces, meningeal enhancement, prominent choroidal fissure, and parahippocampal cysts [9]. However, early cryptococcosis cannot be detected with radiological imaging. That is to say, we cannot perform a prompt treatment at an early stage. Recently, advancements in controlling the surface shape/morphology of gold nanomaterials have demonstrated the great capability to engineer their localized surface plasmon resonance [10, 11]. Herein, we investigated a kind of nanoscale gold material called gold nanorods (GNRs), which can selectively conjunct with the funguses. In clinical CT imaging, iodinated compounds are the most commonly used contrast medium. However, the atomic number and electron density of gold are much higher than those of iodine. Gold can induce a strong X-ray attenuation, which makes it an ideal candidate for CT contrast agents [7]. By conjugating GNRs with specific antibodies, scientists potentially target and capture images from specific tissues and pathogens [12].

Amphotericin B is a major therapeutic agent for the treatment of cryptococcal disease, which has been deployed since late 1960s [13]. However, the clinical efficacy of amphotericin B is limited, and it exhibits significant nephrotoxicity [14]. The efficacy of present drugs is compromised by toxicity, drug resistance, or an inadequate range of activity [15, 16]. So, new selective therapeutic methods for cryptococcal disease need to be devised. Recently, photo-thermal treatments are extensively used to target and destroy cancer cells, viruses, and bacteria [17–19]. Compared to traditional therapeutic regimes, the mechanism of such therapeutic agents is completely different. Near-infrared (NIR) laser light is an ideal photo-thermal treatment method, which can be absorbed specifically by tissues or materials. Light can penetrate effectively through tissues accompanying minimal damage to normal tissues [20]. GNRs absorb light in the NIR region (650–900 nm), and the absorbed light energy can be converted into thermal energy. Based on this principle, it is an ideal method to combine NIR laser light with GNRs for treatment. Compared to classical photosensitizers, GNRs have several advantageous features: high absorption cross section, high solubility, excellent biological compatibility, hypotoxicity, great light stability, and easy conjugation with target molecules [21]. Several reports have described how GNRs be used for photo-thermal treatments [22–24]. Carpin conducted an experiment in breast cancer cells, which over-expressed HER2 gene and were incubated with anti-HER2-conjugated silica–gold nanoshells. Subsequently, the complexes were irradiated by 808-nm NIR radiation. Compared to the control group, the cells were destroyed [17]. Wang reported that antibody-conjugated GNRs could select target and destroy pathogenic *Salmonella* bacteria when exposed to NIR radiation. There was a highly significant reduction in *Salmonella* cell viability [19].

Herein, we used antibody-conjugated silica-modified gold nanorods to specifically bind *C. neoformans* cells. Furthermore, the cells binding to the complexes can be easily distinguished in CT images. These gold nanoparticles were associated with *C. neoformans* cells via immune conjugation, and photo-thermal lysis caused a significant reduction in cell viability. Our study confirmed a brand new option for the diagnosis and photo-thermal therapy of *C. neoformans* in vitro and provides a possibility for treatment of cryptococcosis safely and effectively.

## Methods

### Materials

Anti-*C. neoformans* antibody was purchased from Meridian Life Science (Memphis, TN, USA). Chloroauric acid (HAuCl_4_·3H_2_O) was obtained from Sigma (St. Louis, MO, USA). Silver nitrate (AgNO_3_), tetraethylorthosilicate (TEOS), 3-aminopropyltrimethoxysilane (APTS), cetyltrimethyl ammonium bromide (CTAB), sodium borohydride (NaBH_4_), 1-ethyl-3-(3-dimethyl aminopropyl)-carbodiimide (EDC), poly(sodium-4-styrenesulfonate) (PSS), and ascorbic acid were obtained from J & K Chemical Limited (China). All the above chemicals were used without any further purification. Deionized water (Millipore Milli-Q grade) with a resistivity of 18.2 MΩ cm was used in all the preparations.

### Synthesis of Antibody-Conjugated Silica-Modified Gold Nanorods

**Fig. 1:**
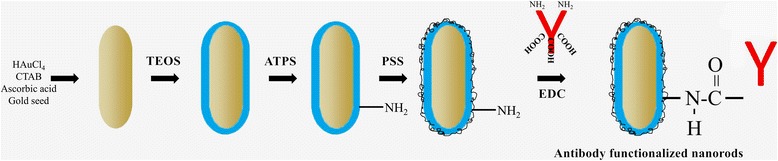
Synthetic procedure of GNR-SiO_2_-Ab

In a typical experiment, GNRs were synthesized according to the seed-mediated template-assisted protocol [25–27]. Synthetic pathway for making the antibody-conjugated silica-modified gold nanorods (GNR-SiO_2_-Ab) is illustrated in Fig. 1. Twenty-five milliliters of the GNR solution was centrifuged at 12000 rpm for 15 min. The supernatant, containing mostly CTAB molecules, was removed, and the precipitate was resuspended in 20 mL anhydrous ethanol adjusted to pH 10 with 20 μL of 28% ammonia. After the system was sonicated, TEOS of 5 mL (10 mM) was added and then the entire system was vigorously stirred for 24 h. Silica-coated GNRs were collected by centrifugation at 4000 rpm for 30 min and were washed three times with water and twice with ethanol. The obtained purified GNR-SiO_2_ samples were redispersed into 10 mL ethanol for further experiment [28]. Subsequently, 10 mL APTS were added to form a mixed solution and allowed to react under refluxing at 60 °C for 1 h. The resultant was washed with deionized water for five times and dried at 60 °C for 3 h in a vacuum oven to obtain the GNR-SiO_2_-NH_2_. The resultant was further coated with a polymer (PSS) by a layer-by-layer technique providing accessible amine groups to solvent [29]. These amine-terminated nanorods were allowed to react with the carboxylic acid of purified antibodies for 12–16 h in the presence of EDC, a water-soluble carbodiimide that promotes amide bond formation between the carboxylic acid and primary amine [30]. Following incubation, the nanorod-antibody complexes were purified by centrifugation and resuspended in PBS [31].

### Characterization of GNR-SiO_2_-Ab

The size and morphology of GNR and GNR-SiO_2_-Ab were characterized using transmission electron microscopy (TEM; Tecnai G2 spirit Biotwin, FEI, USA), operating at an accelerating voltage of 120 kV [20]. UV-vis spectra was measured at 20 °C with a UV-visible spectrophotometer (Shimadzu UV-2450, Shimadzu, Japan) equipped with a 10-mm quartz cell, where the light path length was 1 cm. The 200- to 1000-nm wavelength was scanned, since it includes the absorbance peaks of the GNRs, anti-*C. neoformans* antibody, and GNR-SiO_2_-Ab. The GNR-SiO_2_-Ab was incubated at 4 °C for 2 and 4 weeks. The 200- to 1000-nm wavelength was scanned at the two time points.

### Hounsfield Units of GNR-SiO_2_-Ab Measurement

The aqueous solution of GNR-SiO_2_-Ab with different concentration in the range of 0.04–4 mg/mL was directly detected using a Philips Brilliance 64 CT scanner (Philips Healthcare, Best, The Netherlands). The attenuation values were obtained from the CT imaging software.

### Attachment of *C. neoformans* to GNR-SiO_2_-Ab

*C.neoformans* type A H99 strain was obtained from Shanghai Key Laboratory of Molecular Medical Mycology (Shanghai Changzheng Hospital, Second Military Medical University, Shanghai, China). Funguses were allowed to incubate with the GNRs and antibody-nanorod complexes for 1 h prior to prepping for TEM analysis. Images were collected on a TEM instrument (Tecnai G2 spirit Biotwin, FEI, USA) operating at an accelerating voltage of 120 kV.

### In Vitro CT Scanning of Fungus-Antibody-Nanorod Complexes

The materials and funguses were divided into three groups, which include fungus group (N), GPR-SiO_2_-Ab group (G), and GPR-SiO_2_-Ab-attached *C. neoformans* group (G+N). The concentration of GNR-SiO_2_-Ab aqueous solution above was 4 mg/mL. For in vitro CT imaging, the solutions of the three groups were prepared in 1.5-mL sterile Ep tubes. All CT scans were performed using the above CT system.

### In Vitro Photo-Thermal Therapy Effects

*C. neoformans* cells incubated with and without GNR-SiO_2_-Ab were exposed to NIR laser (LWIRL 808, Laserwave Ltd., China) irradiation for 5 min, with a wavelength of 808 nm and an intensity of 30 mW (4 W/cm^2^). Images were collected on a TEM instrument (Tecnai G2 spirit Biotwin, FEI, USA) operating at an accelerating voltage of 120 kV. After irradiating, cells were incubated for 2 h at 37 °C in the dark. *C. neoformans* cells incubated with and without GNR-SiO_2_-Ab were designed as control groups. Cell viability was determined by performing CellTiter-Glo® luminescent cell viability assay (Promega Corporation, Madison, WI, USA) in accordance with the manufacturer’s instructions [28]. This particular cell viability assay was a homogeneous method, which could determine the number of viable cells. The luciferase-catalyzed reaction between luciferin and ATP was used for the synthesis of metabolically active cells. All the experiments were repeated six times, and their mean values were determined.

### Statistical Analysis

All analyses were performed using SPSS version 13.0 (SPSS, Inc., Chicago, IL, USA). Data are expressed as mean ± SD. *P* value of less than 0.05 was taken to indicate statistical significance. All figures shown in this article were obtained from more than three independent experiments with similar results.

## Results

### Synthesis and Characterization of GNR-SiO_2_-Ab

**Fig. 2:**
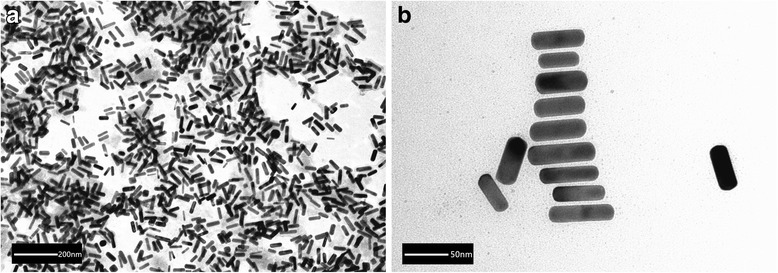
**a**, **b** TEM image of GNR-SiO_2_-Ab. The nanorods presented a rod-like appearance. The shape or size of GPRs did not change when they were conjugated with anti-*C. neoformans*

A method for silica-coated GNRs with TEOS as silica source and APTS as coupling agent has been previously reported [30]. The shape or size of GPRs did not change when they were conjugated with anti-*C. neoformans*. Figure 2 shows the TEM image of GNR-SiO_2_-Ab. These nanoparticles are 18.48 ± 2.39 nm in width and 57.56 ± 4.57 nm in length.

### Spectroscopic Property and Stability of GNR-SiO_2_-Ab

**Fig. 3:**
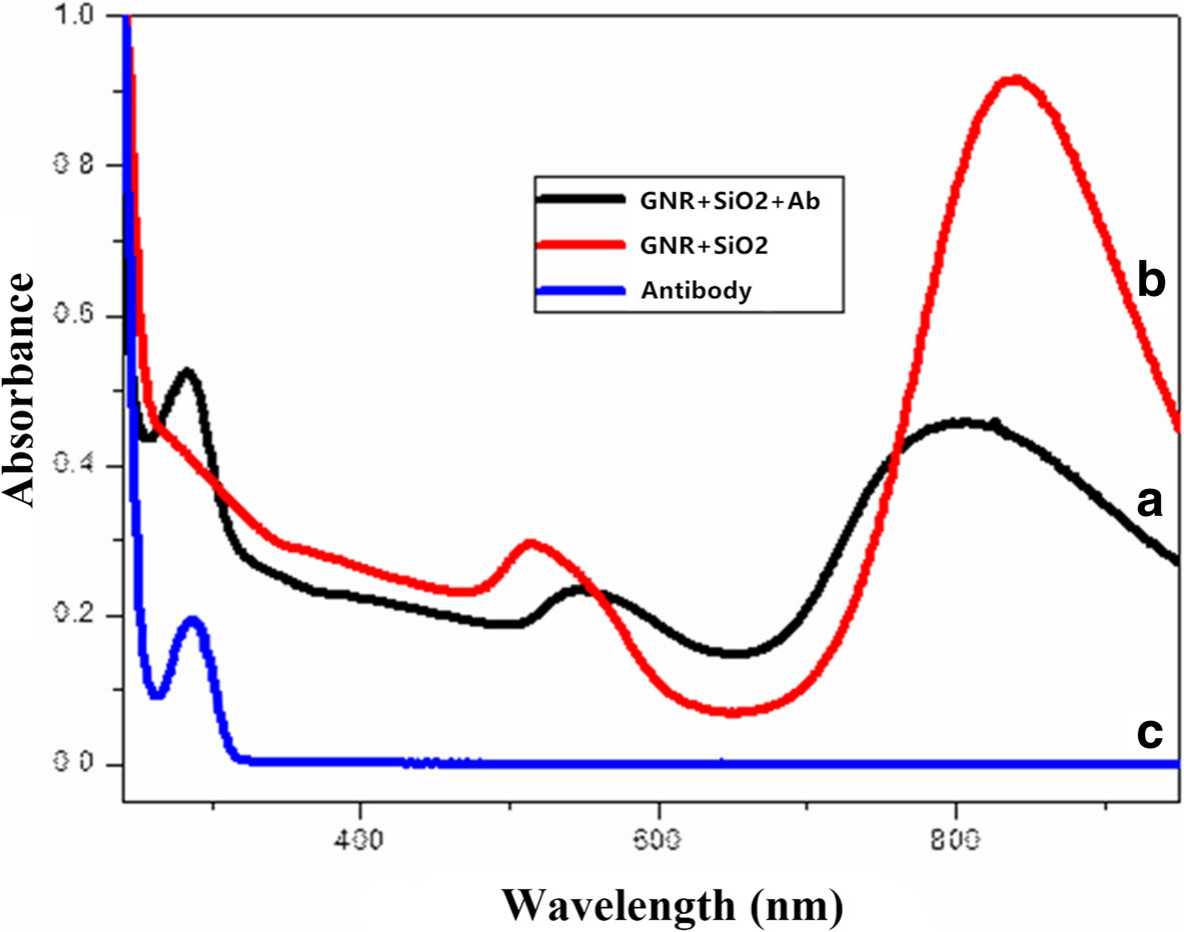
Absorption spectra of: GNR+SiO_2_+Ab (A), GNR+SiO_2_ (B), and anti-*C. neoformans* antibody (C). The GNR-SiO_2_ has two absorption bands, a weak transverse surface plasmon resonance wavelength (TSPRW) around 520 nm and a strong longitudinal surface plasmon resonance wavelength (LSPRW) around 808 nm. After being conjugated with antibodies, the TSPRW and LSPRW of GNR-SiO_2_-Ab are 540 and 835 nm, respectively

Regarding the photo-physical property of GNR-SiO_2_-Ab, Fig. 3 shows the absorbance spectra of GNR-SiO_2_, GNR-SiO_2_-Ab, and anti-*C. neoformans* antibody. The spectrum of GNR-SiO_2_ shows that GNR-SiO_2_ has two absorption bands, a weak transverse surface plasmon resonance wavelength (TSPRW) around 520 nm and a strong longitudinal surface plasmon resonance wavelength (LSPRW) around 808 nm. After being conjugated with antibodies, the TSPRW and LSPRW of GNR-SiO_2_-Ab are 540 and 835 nm, respectively. In a comparison between the spectrum of the antibody and GNR-SiO2-Ab, both of them have the same special peak at around 280 nm. This result proves that the anti-*C. neoformans* antibody was successfully conjugated with GNR-SiO_2_. After incubating at 4 °C for 2 weeks, the TSPRW and LSPRW of GNR-SiO_2_-Ab are 540 and 835 nm, respectively. And the same data was observed at 4 weeks. The TSPRW and LSPRW of GNR-SiO_2_-Ab did not change after incubating for 4 weeks. It confirmed the stability of GNR-SiO_2_-Ab.

### Hounsfield Units of GNR-SiO_2_-Ab Measurement

**Fig. 4:**
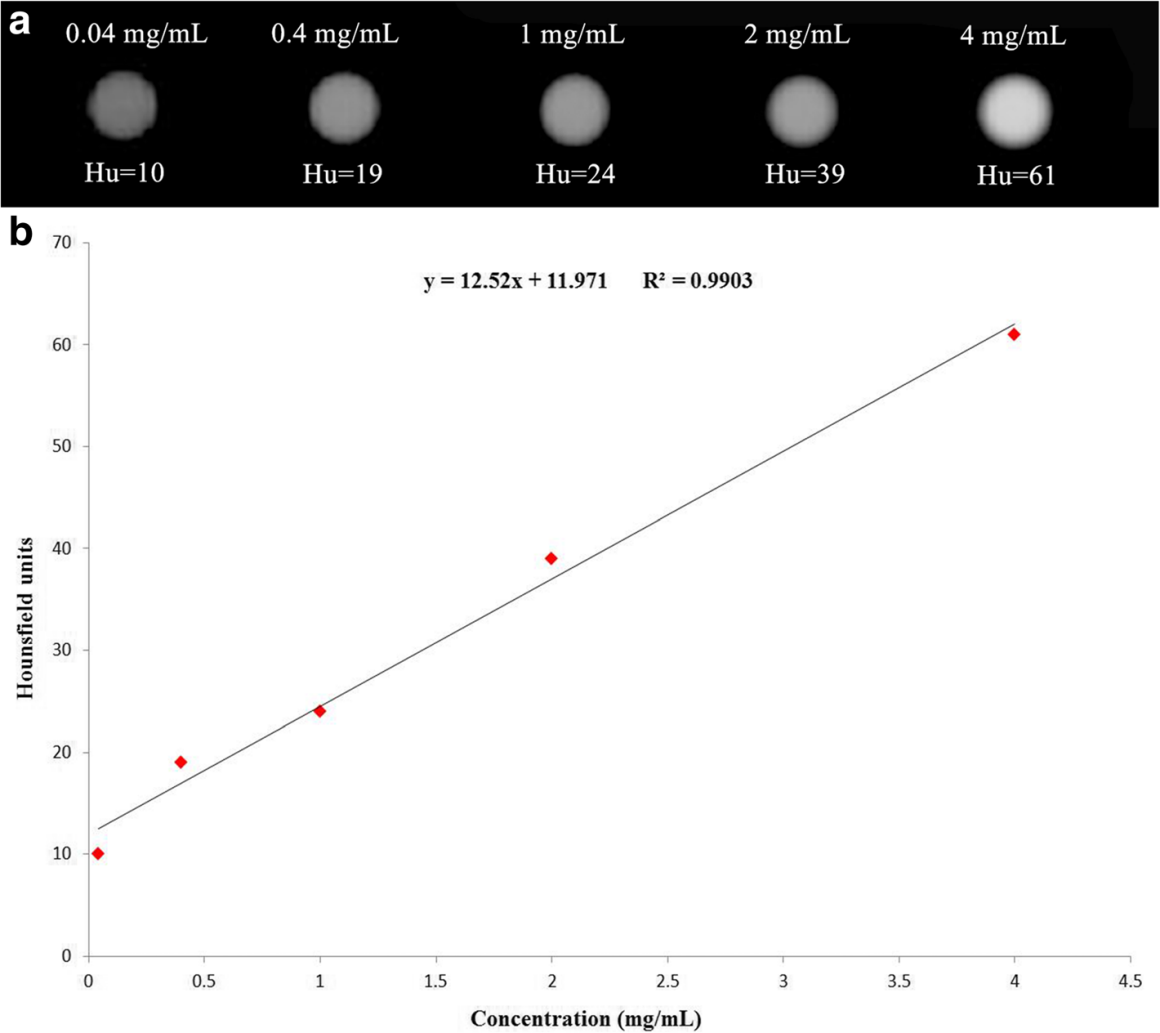
Hounsfield units of GNR-SiO_2_-Ab. **a** In vitro CT images of GNR-SiO_2_-Ab suspended in PBS. The concentration (mg/mL) in each sample is provided at the top of the respective image. **b** CT attenuation plot of GNR-SiO_2_-Ab at various concentrations in the range from 0.04 to 4 mg/mL

The Hounsfield units (Hu) of GNR-SiO_2_-Ab as evaluated by a clinical CT. Figure 4 displays the CT images in the range of 0.04–4 mg/mL of GNR-SiO_2_-Ab. As the concentration of GNR-SiO_2_-Ab increased, the CT signal intensity continuously increased. As shown in Fig. 3, Hu as a function of GNR-SiO_2_-Ab concentration display a well-correlated linear relationship (*R*^2^ = 0.9903), described by the following typical equation: *y* = 12.52*x* + 11.971. These results suggest that GNR-SiO_2_-Ab has a potential application as a positive X-ray/CT imaging contrast agent.

### Attachment of *C. neoformans* Cells to GNR-SiO_2_-Ab

**Fig. 5:**
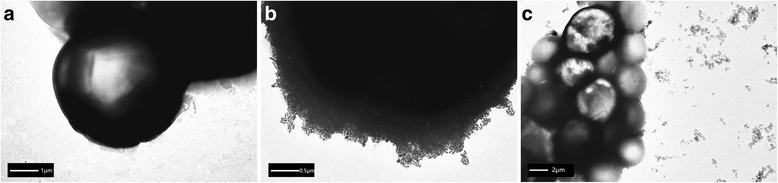
TEM images illustrate the interaction between GNR-SiO_2_-Ab and *C. neoformans* cells. **a** TEM image of *C. neoformans* cells. **b** TEM image of fungus-antibody-nanorod complexes. **c** TEM image of *C. neoformans* cells incubated with GNR-SiO_2_

TEM images show the morphological feature of *C. neoformans* cells and fungus-antibody-nanorod complexes. These cells have a diameter ranging from 2 to 20 μm. Figure 5a displays the TEM image of *C. neoformans* cells, which is surrounded by a polysaccharide capsule. These cells were 4–6 μm in diameter without binding any structures. As shown in Fig. 5b, the *C. neoformans* cells are covered by lots of aggregated GNR-SiO_2_-Ab, after incubating with the antibody-nanorod complexes. We incubated the fungus cells with GNR-SiO_2_ in order to explore whether GNR-SiO_2_ was attached to *C. neoformans.* Our results indicated that GNR-SiO_2_ were scattered as shown in Fig. 5c. Our study shows that *C. neoformans* cells can conjugate with GNR-SiO_2_-Ab selectively.

### In Vitro CT Scanning of Fungus-Antibody-Nanorod Complexes

**Fig. 6:**
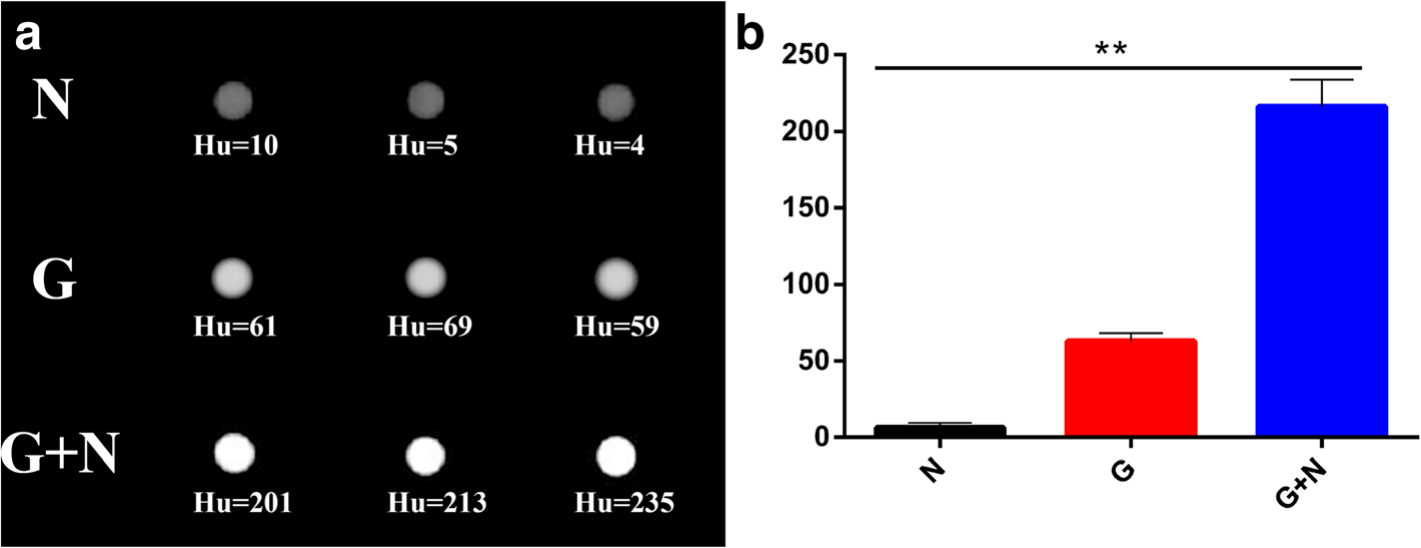
**a**, **b** In vitro CT scanning of different groups. The values of the G+N group were significantly higher than those of the G and N groups. Furthermore, X-ray attenuation values of the G group were significantly higher than those of the N group

We performed quantitative analysis of CT signal intensity via the manufacturer’s standard display program (Philips portal, Philips Healthcare, Best, The Netherlands). Figure 6 depicts X-ray attenuation values of the three groups. The values of the G+N group were significantly higher than those of the G and N groups. Furthermore, X-ray attenuation values of the G group were significantly higher than those of the N group. This result concurs with the findings of previous literature [31].

### In Vitro Effects of Photo-Thermal Therapy

**Fig. 7:**
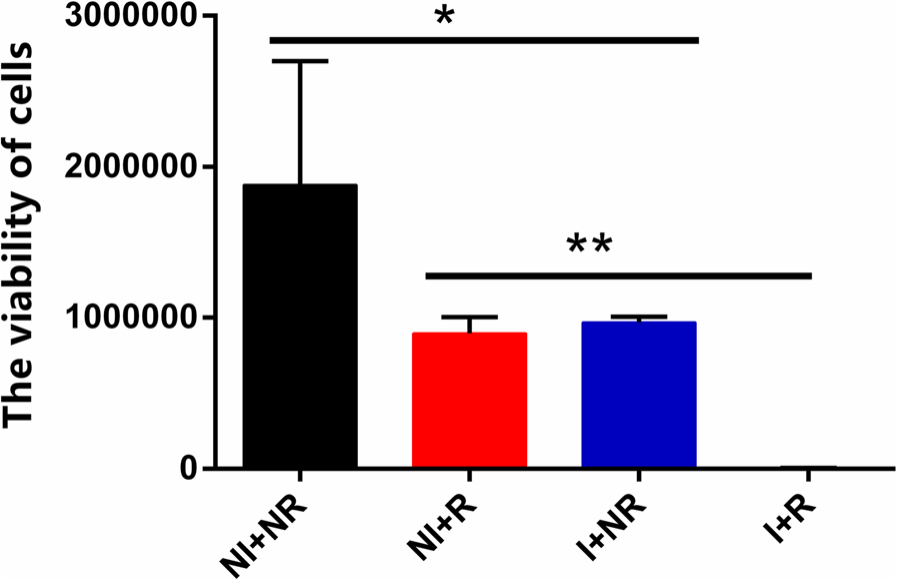
Viability of cells that were treated differently. Cells without irradiating had greater viability than NIR-irradiated cells (*P* < 0.05). Moreover, viability of funguses was higher than that of cells conjuncted with GNR-SiO_2_-Ab after NIR irradiating (*P* < 0.05). In addition, cells had higher viability than fungus-antibody-nanorod complexes (*P* < 0.05)

**Fig. 8:**
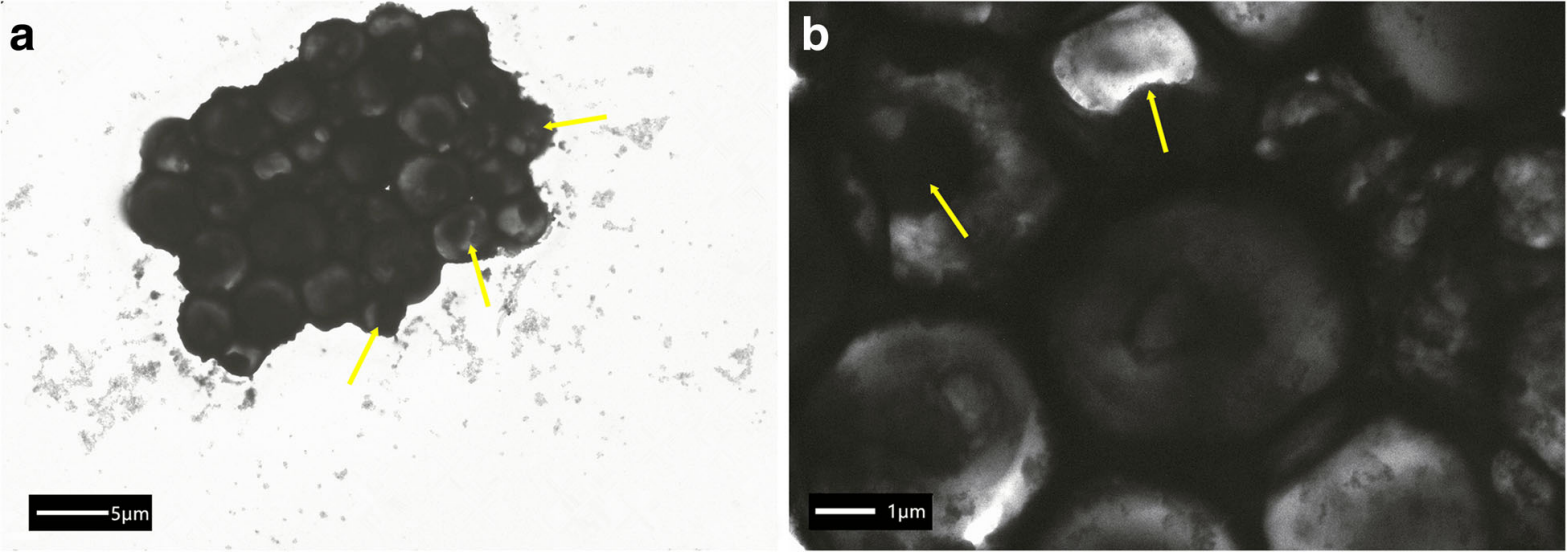
**a**, **b** TEM images demonstrate photo-thermal damages of *C. neoformans* cells that were conjuncted with GNR-SiO_2_-Ab. The cells displayed atrophic, irregular, and collapsed appearances. The characteristic polysaccharide capsule was damaged

We evaluated cell’s viability by performing cell viability assay in a CellTiter-Glo®luminescent instrument. Cells without irradiating had greater viability than NIR-irradiated cells (*P* < 0.05). Moreover, viability of funguses was higher than cells conjuncted with GNR-SiO_2_-Ab after NIR irradiating (*P* < 0.05). In addition, cells had higher viability than fungus-antibody-nanorod complexes (*P* < 0.05). Figure 7 clearly illustrates the variation in the viability of *C. neoformans* cells with different treatments. After irradiation, *C. neoformans* cells suffered photo-thermal damages and the normal structure of the cells were destroyed. As shown in Fig. 8, the cells displayed atrophic, irregular, and collapsed appearances. The characteristic polysaccharide capsule was damaged.

## Discussion

Silica has lots of advantages over polymer [32]. The preparative processes involved are quite facile, and the thickness of a silica shell can be turned to the desired size and porosity. Furthermore, silica is extremely stable and has biocompatibility, has no swelling or porosity changes with a change in pH, and is not vulnerable to microbial attack. In addition, silica has ease of surface modification with a variety of functional groups using silane chemistry and commercially available organosilicon reagents for biotargeting. Silica-coated GNRs retain the superior optical properties of GNRs and can improve their thermal stability under high-energy irradiation. In our study, after being coated with silica and conjugated with anti-*C. neoformans* antibody, GNRs both exhibited a red shift in the surface plasmon resonance peak, due to the increase in the refractive index of the surrounding medium [32, 33]. The results also indicated that the size of the sample becomes bigger and bigger after modification and conjugation. These data indicated that we successfully conjugated gold nanoparticles with an anti-*C. neoformans* antibody. However, we cannot exclude the possibility that the GNR-SiO_2_ wound possesses some special power to conjugate cells after bonding with the antibodies, and we will perform a further study in the future. In this study, we successfully attached GNR-SiO_2_-Ab to the cell capsule through a simple antigen-antibody reaction. In addition, we successfully ensured that our complexes targeted antigens on the cell capsule.

GNRs have gained widespread attention over the last decade. Hainfeld et al. [34] firstly reported that GNRs can be used as an X-ray contrast agent. GNRs confer several advantages over iodinated molecules, a conditional contrast agent. Due to a high atomic number and electron density, GNRs exhibit a relatively high X-ray attenuation coefficiency. The atomic number and electron density of gold (79 and 19.32 g/cm^3^, respectively) are higher than those of iodine (53 and 4.9 g/cm^3^) [7]. Iodine as an X-ray contrast agent has a lot of serious side effects, such as nephrotoxicity and severe allergic reactions. However, GNRs persist in the body much longer than iodine contrast agents, which means there is enough time to observe the images. Furthermore, GNRs can target cancer cells, viruses, and bacteria, via surface functionalization with a variety of molecules, such as peptides or antibodies. Reuveni et al. [31] have shown that several different types of molecules can be attached to the surface of GNRs. In this study, the CT signal intensity continuously increased, together with the increased concentration of GNR-SiO_2_-Ab, resulting in brighter images. GNR-SiO_2_-Ab exhibited significant positive potential as X-ray/CT imaging contrast agents, as well as GNRs. The X-ray absorption of gold nanorods remained unaffected, even with surface modification. These data indicate that the X-ray attenuation properties of GNR-SiO_2_-Ab did not change significantly as a result of surface modification. This concurs with findings reported in previous literature [35–37]. Surface functionalization is a powerful tool which enables passive or active targeting of GNRs to a specific site of interest. In our study, we successfully attached GNR-SiO_2_-Ab to the capsules of *C. neoformans*. In addition, we determined whether these antibody-conjugated particles could be used as nanoprobes while conducting targeted CT imaging of *C. neoformans* cells in vitro. We observed that CT images of *C. neoformans* cells dispersed in PBS appeared quite similar to images derived from *C. neoformans* cells dispersed in water. However, it is difficult to distinguish the images of fungi from soft tissues. An antibody can induce a much greater aggregation of GNRs by binding to the surface of *C. neoformans* cells resulting in a much higher attenuation values than ever. Thus, we can successfully achieve distinguishable X-ray attenuation of funguses. Based on our results, the detection of *C. neoformans* by CT imaging could be achieved and bring novel opportunities in diagnostics.

GNRs have been widely employed for tumor photo-thermal therapy [22, 38, 39]. Our study indicates that GNR-SiO_2_-Ab could be a selective tool to destroy *C. neoformans* cells. Our results confirmed that the cell membrane of *C. neoformans* cells suffered irreparable and badly destruction after being irradiated by NIR. In addition, the viability of cells reduced significantly compared to the untreated cells. These results indicated that NIR radiation alone causes the death of *C. neoformans* cells. However, the viability of cells incubated with GNR-SiO_2_-Ab had also been depressed. In cells incubated with GNR-SiO_2_-Ab and subjected to NIR irradiation, the viability of cells reduced significantly compared to other groups. We make sure that the GNR-SiO_2_-Ab conjugated with the funguses selectively and improved the effects of the NIR radiation. GNR-SiO_2_-Ab has the capability of selective photo-thermal therapy effects on the *C. neoformans* cells. The mechanism of effect has not been reported before. We speculate that disruption in cell membrane was probably induced by irradiation-induced cell death. Norman et al. [18] reported that viability of *Pseudomonas aeruginosa* was reduced significantly when this species was exposed to irradiation and bound with gold nanorods, which were covalently conjugated with specific antibodies. These cells also showed areas of badly disrupted cell membrane with irreparable damage, which was caused by exposure to NIR irradiation. When nanoparticles were exposed to NIR radiation, the cell membrane was damaged owing to several factors, including nanoparticle explosion, shock waves, bubble formation, and thermal disintegration [40].

In this study, the death or reduced activity of *C. neoformans* cells occurred when the cell membrane was disintegrated and destroyed by thermal energy. However, further studies should be conducted to confirm this hypothesis. *C. neoformans* cells are damaged by the following factors: localized increases in temperature, nanoparticle explosion, shock waves, bubble formation, and thermal disintegration that is caused by NIR radiation. In particular, *C. neoformans* cells were substantially damaged when exposed to only NIR radiation. There are two possible reasons to explain how targeted GNR-SiO_2_-Ab stimulates NIR radiation by eliciting photo-thermal destruction of *C. neoformans* cells. One possibility is that in the capsule of *C. neoformans* cells, the targeted GNR-SiO_2_-Ab induces a localized increase in temperature. The second possibility is that owing to an antigen-antibody reaction between GNR-SiO_2_-Ab and the capsule of *C. neoformans* cells, there are structural changes in the cell wall and capsule. Owing to such changes, cells would be more sensitive towards photo-thermal treatment [41]. Previous studies have confirmed the low toxicity of the gold nanorods [22, 42, 43], and further studies will be required to investigate the effect of photo-thermal therapy in vivo. The most regrettable of our study is that we did not discuss the loading capacity of GNR-SiO_2_-Ab. Further study will concentrate on the relationship between the loading capacity of GNR-SiO_2_-Ab and the photo-thermal effect.

## Conclusions

We successfully fabricated GNR-SiO_2_-Ab, which was targeted towards *C. neoformans* cells. These specific antibody-conjugated gold nanorods enhanced the X-ray attenuation of *C. neoformans* cells in CT images. Our results indicated that immune GNRs, which were mediated by antibodies, increased the effects of NIR-induced photo-thermal therapy in *C. neoformans* cells. Furthermore, GNR-SiO_2_-Ab allowed easy manipulation and minimally invasive procedures in the diagnosis and treatment of *C. neoformans* infections, focusing on the potential clinical application of this approach.
